# On the mechanism of the cholesterol lowering ability of soluble dietary fibers: Interaction of some bile salts with pectin, alginate, and chitosan studied by isothermal titration calorimetry

**DOI:** 10.3389/fnut.2022.968847

**Published:** 2022-09-29

**Authors:** Michele Massa, Carlotta Compari, Emilia Fisicaro

**Affiliations:** ^1^Department of Maternal Infantile and Urological Sciences, Sapienza University of Rome, Rome, Italy; ^2^Department of Food and Drug, University of Parma, Parma, Italy

**Keywords:** cholesterol-lowering ability, soluble dietary fiber, alginate, pectin, chitosan, bile salt, soluble dietary fiber-bile salt interaction, functional foods

## Abstract

Reducing high blood cholesterol is an important strategy to decrease the chances of a cardiovascular disease occurrence, the main cause of mortality in western developed countries. Therefore, the search for an alternative therapeutic or preventive approach being natural, biocompatible, and not toxic is still more relevant than ever. This need is particularly felt in Pediatrics for treating childhood hypercholesterolemia, due to statins interference in the production of steroid hormones in prepuberal children. Notwithstanding the general acceptance of the healthy role of the fibers in the diet, the mechanism underlying the cholesterol-lowering ability of soluble fibers is still under discussion. Therefore, we started a systematic study of the binding ability of some soluble dietary fibers (SDF) originated from different natural sources toward selected bile salts (BS) by isothermal titration calorimetry (ITC). Here we report the results of our ITC studies on the interaction of alginate, pectin and chitosan with sodium cholate (NaC), sodium deoxycholate (NaDC), sodium taurocholate (NaTC) and sodium taurodeoxycholate (NaTDC). Thermodynamic data on the micelle formation process of the above bile salts, as a premise to the study of their binding ability to the SDF, are also reported. Alginate does not show specific binding interaction with BS, while pectin shows a strong exothermic bond with NaDC in monomeric form. Chitosan, positively charged and soluble only at low pH, shows strong exothermic interactions with NaTC and NaTDC (soluble at pH = 3 in acetate buffer) with precipitate formation. For NaTC, the exothermic peak starts at about 5 mM. At this concentration NaTC bound on the fiber reaches locally the cmc value and micelles start forming on the fiber inducing its conformational change. For NaTDC the same process occurs at much lower concentrations, due to lower cmc, and with a greater quantity of heat involved. The first set of results here presented shows that for some SDF the binding of BS could be an important mechanism in cholesterol lowering but not the only one. The information here presented could be a starting point for the design of optimized functional foods with high cholesterol lowering ability.

## Introduction

It is well known that cardiovascular diseases (CVD) are the main cause of mortality in western developed countries and that a high serum level of LDL cholesterol and obesity are considerable risk factors (1). Reducing high blood cholesterol is an important strategy to decrease the chances of a CVD occurrence. This can be obtained in adults by the administration of cholesterol lowering drugs, such as fibrates and statins (2, 3), but the pharmacological treatment is not without the risk of adverse side effects and intolerance. Adherence to pharmacological treatment is largely suboptimal and LDL targets are not achieved in up to 80% of high-risk patients (4). Therefore, the search for an alternative therapeutic or, better, preventive approach being natural, biocompatible, and not toxic is still more relevant than ever. This need is especially felt in the western world, due to the general aging of the population. The cornerstone of lipid-lowering therapy remains healthy lifestyle but facing this problem with the modification of the dietary habits is very challenging. Moreover, the pediatric population deserves a special mention as it is well known that, since childhood, hypercholesterolemia is a major risk factor for early cardiovascular disease and appropriate therapy could prevent the progression of atherosclerotic vascular changes (5). Childhood hypercholesterolemia can be either primary, due to hereditary disorders or secondary to other disease such as obesity, diabetes mellitus and nephrotic syndrome. Dietary treatment and lifestyle approach represents the first line treatment of childhood hypercholesterolemia. Nutrition education, for an efficient chronic illness prevention, must start from the childhood, being well known, above all, that childhood obesity has reached an epidemic level in developed countries. Overweight and obesity in childhood have significant impact on both physical and psychological health (6), without considering that an obese child is more likely to become an obese adult. Controversies among experts still exist on pharmacological treatment of pediatric hypercholesterolemia. Statins are the first line therapy in children older than 8 years (7) but concerns about long term safety, especially because their interference in the production of steroid hormones in prepuberal children, limits their use (8). Therefore, special attention is paid to the recommendation of a correct diet starting from early life, and, more recently, to the formulation of functional foods, able to control serum cholesterol. The supplementation of soluble fibers has been shown to enhance the cholesterol-lowering effect of diet (9). Several studies have pointed out the role of soluble dietary fibers (SDF), generally present at high levels in fruits and vegetables (10–12). The relevance of fibers in the diet not only of the adults but also in children over the age of 3, has been recognized by the American Health Foundation, recommending a consumption of 5 plus child’s age, in grams of fibers (12). The cholesterol-lowering effect of SDF has been known for over 40 years (13). The health effects of soluble fibers consumption, confirmed by many epidemiological studies, have been recently reviewed (13–16). Consumption of SDF could provide many health benefits, between them reduced lipid levels with reduced cardiovascular disease risk, control of blood glucose, lowering of blood pressure, improved immune function, reduced inflammation, weight loss (16). Therefore, the understanding of the precise chemical mechanism underlying their activity would be of paramount importance in order to shed light and, possibly, optimize their effect.

Bile salts are natural surface-active molecules playing an important role in lipid digestion. Synthesized in the liver from cholesterol and then secreted in the duodenum, they are in the largest part reabsorbed within the gastrointestinal tract and sent back to the liver. Some dietary fibers can interfere with their reabsorption, probably through a specific binding (13–17). This binding could influence the emulsifying ability of bile salts and impact on the digestion and transport of lipids, reducing their availability. Other mechanisms have been proposed to account for the experimental evidence of healthy effects of SDF, as, for instance, the repression of digestive enzymes or the prevention of lipids absorption through the intestine wall by increasing the barrier properties of the unstirred layer between BS micelles and intestinal absorptive cells or by forming a local matrix entrapping BS micelle (16). Notwithstanding the general acceptance of the healthy role of the fibers in the diet, a well-founded mechanism of blood cholesterol reduction by each SDF is still lacking. Between the proposed biological mechanisms, that one having the greatest acceptance is the ability of SDF to bind bile salts, preventing their reabsorption and so leading the liver to consume hepatic cholesterol by synthesizing new bile salts to maintain the homeostasis. In fact, the synthesis of bile salts is the major route by which the hepatic cholesterol is eliminated (11, 18). The bile is constituted by different kinds of bile salts: cholic, chenodeoxycholic, and deoxycholic acids are the most abundant (about 95%). Every day liver transforms 200–400 mg of cholesterol in “primary” bile acids (cholic and chenodeoxycholic acids, in ratio 2:1). Primary acids, released from the liver, are conjugated with the amino group of glycine (glycocholic and glycochenodeoxycholic acids) and of taurine (taurocholic and taurochenodeoxycholic acids) in ratio 3:1. Not absorbed primary acids are converted by bacteria in secondary bile acids (deoxycolic and lithocolic acids). Moreover, there are additional secondary bile acids, such as ursodeoxycholic acid. On the other hand, dietary fibers originate from different natural sources and differ by kind, number, bond of the subunits constituting their molecular frame (17).

A systematic study of the bile salts binding ability of the different SDF is lacking in the literature. Are really able the different SDF to bind bile salts? Are the different bile salts bound in the same amount? Is there a selectivity of the different SDF for a given bile salt? To begin to answer to the above questions, we started to study the interaction between some selected SDF and bile acids by isothermal titration calorimetry (ITC). This information is useful to design new functional foods containing a pool of fibers with the best performances in reducing cholesterol.

## Materials and methods

### Materials

Sodium cholate hydrate (NaC), sodium taurocholate hydrate (NaTC), sodium deoxycholate (NaDC), and sodium taurodeoxycholare (NaTDC) from Sigma-Aldrich were used as received. Solutions at concentration 40, 50, 200 mM, depending on the experiment, were prepared using boiled doubly distilled water or in 100 mM acetate buffer at pH = 3, in which only NaTC and NATDC are soluble.

Alginic acid sodium salt from brown algae was supplied by Sigma-Aldrich. Low methoxyl pectin from citrus peels (CU701, DE = 38%) was provided by Herbstreit & Fox Neuenbuerg (Germany). Chitosan, (batch 34480004, 91.1% deacetylated) was supplied by ACEF, Fiorenzuola d’Arda (PC, Italy). All the SDF were used as received. 1% w/v SDF solutions were prepared by weight and stirred overnight at a constant rate.

### Isothermal titration calorimetry

ITC measurements were carried out on a CSC model 5300 N-ITCIII isothermal titration calorimeter (Calorimetry Sciences Corporations, USA) at 30°C. BS solution (in water for experiments with alginate and pectin and in 100 mM acetate buffer at pH = 3 for chitosan) was injected (25 injection of 10 μL each) into a 960 μL reaction cell filled with the solvent for studying the micelle formation thermodynamics or with a SDF solution 1 g L^–1^ by a 250 μL syringe with an interval of 600 s between two successive injections. A continuous stirring at 150 rpm was maintained throughout the experiments. NaC dilution experiments were performed from 11° to 40°C. Data were analyzed by the Nano Analyze software from TA instruments, version 1.2.1.0.

## Results and discussion

Bile salts, playing a pivotal role in many physiological processes (17–21), possess surface active properties. They differ from the classical surfactants in the disposition of hydrophilic and hydrophobic moieties. Classical surfactants are composed by a hydrophobic tail (generally a hydrocarbon chain) and by a polar headgroup, whereas bile salts have hydrophilic and hydrophobic surface, arranged on the concave (α) polyhydroxylated and on the convex (β) side of the rigid steroid ring system, respectively. The number and the position of the -OH groups bound to the steroid ring influence the hydrophilic/lipophilic balance of the molecules so determining many tensidic and biochemical properties. For a detailed conformational analysis of the steroid skeleton of the bile salts the reader could refer to Poša (22). For this reason, they form micelles, but their aggregation properties greatly differ from those of classical surfactants, due to a very low aggregation number and a broad micelle formation range (23–26). Moreover, the mean size of their aggregates shows a strong concentration dependence with the formation of primary and secondary micelles, as found also by computer simulation (27). To study the interaction between bile salts and soluble fibers by ITC we must use titrating solutions of bile salts at a concentration much greater than the cmc. Therefore, the design of our binding experiments by ITC needs the evaluation of their dilution heats, including the heat of demicellization, dependent on temperature and generally not negligible. Because the micelle formation process is a typical hydrophobic process, the ln cmc (related to the change in free energy upon micelle formation, Δ*G*_mic_) as a function of temperature has a parabolic trend with a minimum at a temperature at which Δ*H*_mic_ = 0 (28). For a greater accuracy of the ITC binding experiments, the best condition is to carry out the measurements at a temperature equal or near to that of the minimum so that the heat due to the demicellization process, does not interfere too much with the heat of binding. For this reason, we started studying the micellization process of NaC as a function of temperature. After having defined our working temperature, we defined at that temperature the thermodynamics of micelle formation of the bile salts used in this work.

### Micelle formation thermodynamics of bile salts

**FIGURE 1:**
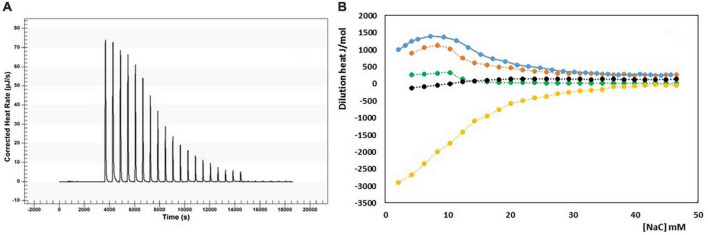
**(A)** Output of the ITC experiment carried out at 11°C (25 injection of 10 μL, each); **(B)** trends of molar dilution heats as a function of NaC concentration in the calorimetric cell, starting from a titrating solution 200 mM: 284.16°K (yellow), 298.16°K (black); 303.16°K (green); 310.16°K (red); 313.16°K (blue).

**FIGURE 2:**
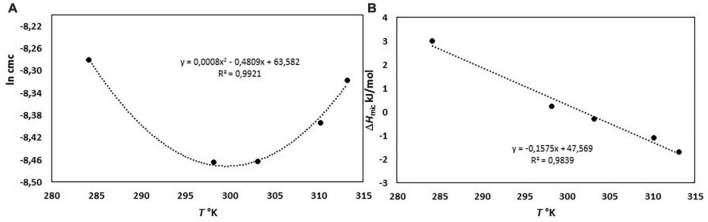
**(A)** ln cmc (expressed as mole fraction) as a function of 1/T and **(B)** the micellization enthalpies as a function of T.

The micellization process of bile salts has been studied from the end of last century by many techniques, recently reviewed by Natalini et al. (25) and Madenci and Egelhaaf (29). Thermodynamic parameter of micellization have been measured both by the dependence of cmc from temperature (29, 30) and by ITC (29–33). We have performed the ITC dilution experiments of a NaC solution 200 mM from 10 to 40°C. At this concentration, NaC is at list 10 times above its cmc and it is found mainly in the form of micelles. By adding the solution in the calorimetric cell containing water, micelles are destroyed, and the heat observed is mainly relative to the process of micelle disruption. Above the cmc region, the heat due to the process of micelles dilution is registered. We have obtained the changes in enthalpy of micellization by applying a pseudo-phase transition model, in which the aggregation process is considered like a phase transition, taking place at equilibrium. The micellization parameters are obtained by extrapolating at the c.m.c. the trends of dilution enthalpies before and after c.m.c. (20–36), and reference therein] where rather sharp changes occur, but they are never discontinuous, especially for surfactants with short hydrophobic chains, i.e., forming small micelles as it is for bile salts. The value of the cmc and Δ*H*_mic_ can both be obtained by an ITC experiment, as clearly described in Olofsson and Loh (36). The cmc value can be derived from ITC measurements in two different ways, or as the concentration where 1% of the surfactants is in micellar form (that is the crossing point between extrapolated initial and linear ascent lines) or the cmc is assumed as the inflection point in the titration curve. When the surfactant micelles have a great aggregation number, both methods give about the same value, but with low aggregation number, as is the case of bile salts, the two methods significantly differ, because the transition between monomers and micelle zone is not sharp but spans a great concentration range. This is the case of bile salts for which a stepwise micellization model has been also proposed (37, 38). For NaC micelles an aggregation number between 4 and 16, depending on the method used for its detection, and a size of its micelles of 1.4 nm (much smaller than that of SDS micelles, notwithstanding the monomers have similar length) has been reported (37). We have chosen, in agreement with the greatest part of the data reported in the literature, to use the inflection point of the titration curve as cmc, notwithstanding its not straightforward detection. In Mukherjee et al. (26), for instance, the inflection point of the curve describing the thermal output as a function of the total detergent concentration has been chosen as the maximum of the first derivative of the curve, disregarding the mathematical condition for the inflection point (first and second derivative of the curve equal to zero). Results from our experiments are reported in Table 1. In Figure 1 is shown, as an example, the output of the calorimetric titration experiment carried out at 30°C and the trends of molar dilution heats as a function of the concentration in the calorimetric cell, starting from a titrating solution 200 mM, for all the temperature examined. It is important to outline that all the experiments use a titrating solution with the same NaC concentration. Therefore, the trends shown in Figure 1 are the trends of apparent molar enthalpies with reference state 200 mM NaC, allowing for the evaluation of the change in micellization enthalpy by a pseudo-phase transition model, all referred to the same standard state. In Figure 2 the values of ln cmc (expressed as mole fraction) as a function of 1/T and of the micellization enthalpies as a function of T are reported. Figure 2A reports the van’t Hoff equation


$${\left\{\delta \,l\,n\,c\,m\,c/\delta \,\left(1/T\right)\right\}}_{P}=\Delta \,H_{\text{mic}}/R$$

**TABLE 1:** Cmc and thermodynamic parameters for micelle formation, obtained from ITC experiments, as a function of temperature for NaC.

*T*°K	Cmc [M]	Δ *H*_mic_ [kJ mol^–1^]	Δ *G*_mic_ [kJ mol^–1^]	Δ *S*_mic_ [J K^–1^ mol^–1^]
284.16	0.0128	3.0	–19.8	80.2
298.16	0.0121	0.3	–20.9	70.9
303.16	0.0122	–0.3	–21.2	69.0
310.16	0.0127	–1.1	–21.6	66.1
313.16	0.0130	–1.7	–21.8	64.0

for the system under study, showing the parabolic trend typical of the hydrophobic processes (28).

The micellization free energy, Δ*G*_mic_ is obtained from a pseudo-phase separation model, the same used for obtaining the micellization enthalpy from ITC data, from the equation


$$\Delta \,G_{\text{mic}}=R\,T\,l\,n\,c\,m\,c$$

where the cmc is expressed as mole fraction, without considering the degree of counterion binding, β as in mass-action model (28, 30). Moreover,


$$\Delta \,S_{\text{mic}}=\left(\Delta \,G_{\text{mic}}-\Delta \,H_{\text{mic}}\right)/T$$

The cmc values and the thermodynamic parameters for the micellization process so obtained are reported in Table 1. They are in quite good agreement with similar data from the literature (30, 31, 37), in the limits of the error intrinsic of the methodology adopted.

**FIGURE 3:**
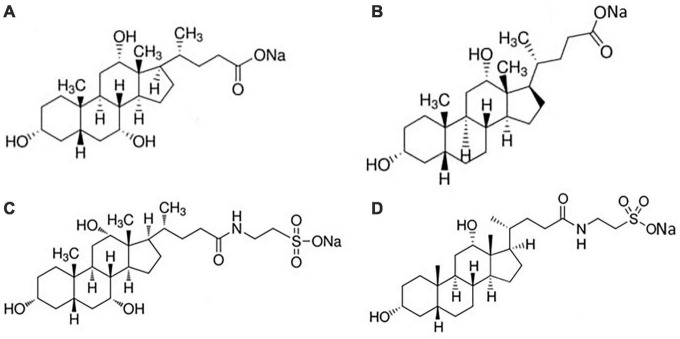
Structures of **(A)** sodium cholate; **(B)** sodium deoxycholate; **(C)** sodium taurocholate; **(D)** sodium taurodeoxycholate.

In Figure 2B the linear correlation between Δ*H*_mic_ and temperature is shown: the slope of the plot is Δ*C*_*p*, mic_, the change in heat capacity upon micellization. It results –157.5 J K^–1^ mol^–1^, lower as absolute value than that reported in Mukherjee et al. (26). If divided by the molar heat capacity of water (Δ*C*_*p*, mic_ = n_*w*_*C*_*p*,w_, where *C*_*p,w*_ = 75.36 J K^–1^ mol^–1^ is the heat capacity of water), yields the number *n*_*w*_ which represents the number of water molecules involved in the formation of the cavity to host the micelle (28). This number results *n*_*w*_ ≈-2. The negative value means that the formation of micelles causes a negative change in the volume of the cavity when the hydrophobic moieties stick together. This number, proportional with the dimensions of solute, is comparable, for instance, with that of decyl-trimethyl ammonium bromide (28). Moreover, Figure 2B show that Δ*H*_mic_ cross the zero value around 30°C, in good agreement with literature data (29, 30). Therefore, we have adopted this temperature (not too much far from body temperature) for our experiments with soluble fibers. At this temperature, we measured the dilution heats for all the BS used in this study, so obtaining their micellization parameters at this temperature (Table 2). In human the unconjugated bile salts are present in very small amounts, at a difference with the bile salts conjugated with taurine and glycine, but their simpler structure could be of great help in the study. For clarity, we report in Figure 3 the structures of the bile salts used in this work: sodium cholate has one OH group more than sodium deoxycholate; sodium taurocholate and sodium taurodeoxycholate derive from the previous ones due to the conjugation of the carboxylic group with the amino-acid taurine. Due to their structure sodium cholate and sodium taurocholate are less hydrophobic than deoxy salts. Results in Table 2 are in agreement with the above observations. We outline that the cmc values of bile salts reported in the literature are scattered and strongly dependent on the methodology used for the evaluation (21–26, 29). As suggested many times in the literature (36–41), the values of cmc from surface tension, for instance, are lower than those from conductivity or fluorescence. Rosen et al. found that the occurrence of premicellar aggregates caused the cmc, as determined by both surface tension and conductivity, to be substantially different in value (41). The hypothesis of the formation of dimers before cmc has also been proposed for bile salts (26, 29, 42). The general trend is that the cmc values of dihydroxy bile salts, NaDC and NaTDC are lower than those of trihydroxy salts, NaC and NaTC (29), perfectly in agreement with our results.

**TABLE 2:** Cmc and thermodynamic parameters for micelle formation, obtained from ITC experiments, for the bile salts under study at 303.16°K.

Compound	Cmc (M)	Δ *H*_mic_ [kJ mol^–1^]	Δ *G*_mic_ [kJ mol^–1^]	Δ *S*_mic_ [J K^–1^ mol^–1^]
NaC	0.0122	–0.30	–21.2	69.0
NaDC	0.0090	–1.50	–22.3	68.6
NaTC	0.0140	–0.50	–20.9	67.2
NaTDC	0.0050	–3.10	–23.5	67.2

### Bile salts interaction with alginate and pectin

Alginate and pectin are both polyuronates, natural polyelectrolytes composed of uronic acids. They have a wide range of applications particularly in food, pharmaceutical, and medicine industry (12, 43, 44). Alginate, produced mainly from brown seaweeds, is formed by β-D-mannuronate (M) and α-L-guluronate units (G), linked *via* (1→4) glycosidic linkages (43). The linear macromolecule is formed by homopolymeric bloks of G and M and by blocks of alternating sequence of G and M (12, 43–46). Pectin, extracted from plant cell walls, is composed by smooth regions of partially methyl esterified α-D-guluronate residues, linked *via* (1→4) glycosidic bonds, and by hairy regions in which other sugars are present (12, 43–45). For a pictorial representation of the structures of the two different soluble fibers the reader should refer, for instance, to Figure 2 of Pinazo et al. (39). To find out experimental evidence of binding between some SF with bile salts we have chosen to use ITC measurements due to the high sensitivity of the method, not requiring pretreatment of the sample. ITC measurements can provide clear information about the presence of specific interactions, also in complex systems as those constituted by dietary fibers. Data reported and discussed in the following are relative to the interaction of pectin and alginate with (a) NaC, (b) NaDC, (c) NaTC, and (d) NaTDC.

**FIGURE 4:**
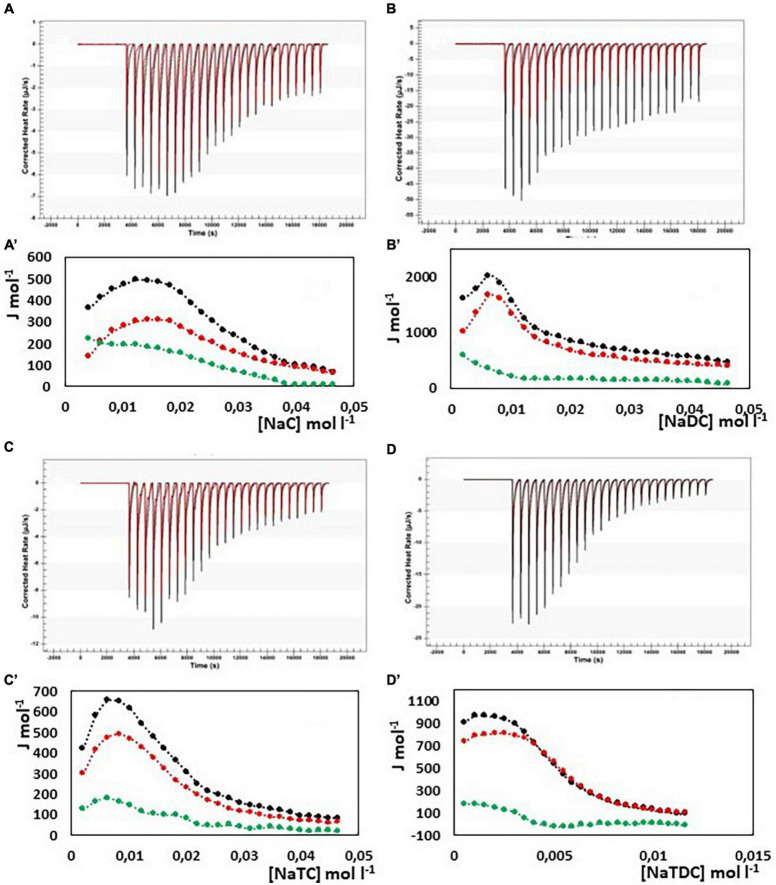
Heat rate (μJ s^–1^) vs. time profiles obtained from 25 injection each of 10 μL into a 960 μL reaction cell filled with an alginate solution 1 g L^–1^ (black) compared with the dilution of bile salt (red) in the same conditions, and (specified by a single quote mark) the dependence of the enthalpy change vs. bile salt concentration in the reaction cell for: **(A)** NaC 200 mM; **(B)** NaDC 200 mM; **(C)** NaTC 200 mM; **(D)** NaTDC 50 mM. Black dots represent the interaction enthalpy between alginate and bile salt, the red dots the dilution enthalpies of bile salt and the green dots their difference.

**FIGURE 5:**
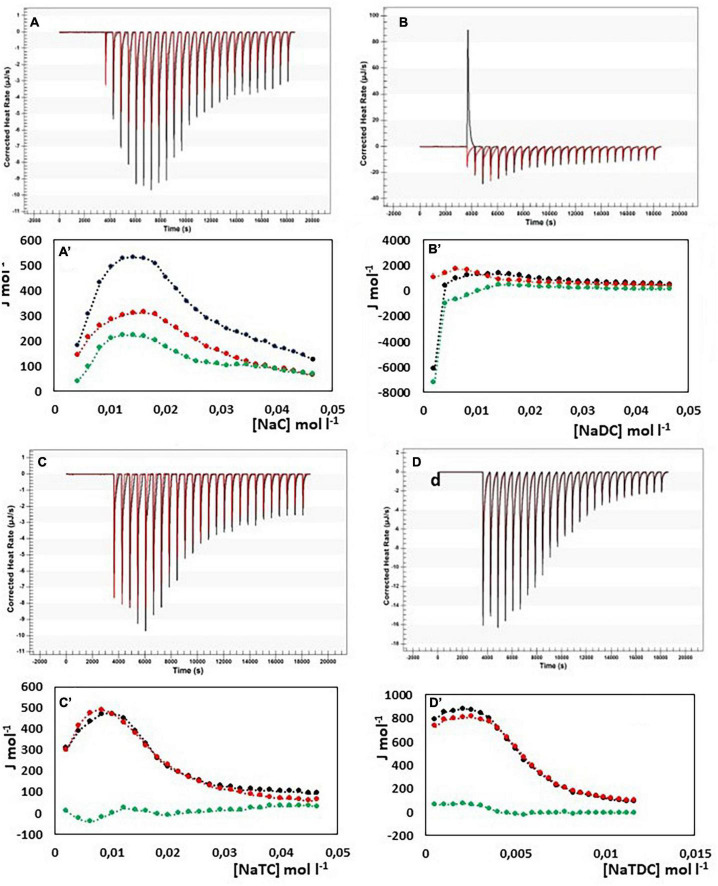
Heat rate (μJ s^–1^) vs. time profiles obtained from 25 injection each of 10 μL into a 960 μL reaction cell filled with a pectin solution 1 g L^–1^ (black) compared with the dilution of bile salt (red) in the same conditions, and (specified by a single quote mark) the dependence of the enthalpy change vs. bile salt concentration in the reaction cell for: **(A)** NaC 200 mM; **(B)** NaDC 200 mM; **(C)** NaTC 200 mM; **(D)** NaTDC 50 mM. Black dots represent the interaction enthalpy between pectin and bile salt, the red dots the dilution enthalpies of bile salt and the green dots their difference.

**FIGURE 6:**
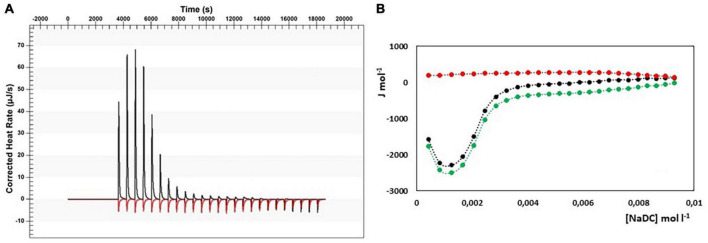
**(A)** Heat rate (μJ s^–1^) vs. time profiles obtained from 25 injection each of 10 μL of NaDC 40 mM into a 960 μL reaction cell filled with a pectin solution 1 g L^–1^ (black) compared with the dilution of NaDC 40 mM (red); **(B)** the dependence of the enthalpy changes vs. NaDC concentration in the reaction cell. Black dots represent the interaction enthalpy between pectin and bile salt, the red dots the dilution enthalpies of bile salt and the green dots their difference.

In Figure 4, the results from ITC experiments concerning the interaction between alginate and the bile salts under consideration are shown. In detail, in Figures 4A–D the output rate in μJ s^–1^ as a function of time, both for bile salt dilution experiment (red) and for the interaction of bile salt with alginate (black) are reported, following 25 injections of 10 μL each of bile salt solution in water and in the 1 g/L alginate solution, respectively. The integration of the peaks allows to obtain the Δ*H* of dilution of bile salts (red dots) and of interaction with alginate (black dots) as a function of bile salt concentration in the calorimetric cell, shown in Figures 4A’–D’. In the figure, the green dots represent the difference between the black and the red curves, this is to say the net interaction heat between alginate and bile salts, because the heat of dilution of the alginate is under the detection limit of the instrumentation and can be disregarded. In the case of alginate, the residual heats (the green dots in Figure 4), i.e., the interaction heat subtracted of the dilution heat of the bile salt, are endothermic and in general quite small and not indicative of a specific interaction with alginate. Below the cmc, where the bile salt is present as monomer, a little greater endothermal output is observed probably due to an adjustment of the electrostatic interaction in solution between the fiber and the dissociated bile salts. Above the cmc, the absence of thermal response suggest that the alginate do not interacts at all with the bile salts in micellar form. The same considerations could be done when the interaction of pectin with NaC, NaTC, and NaTDC is considered (see Figures 5A,C,D). When the pectin solution is titrated with NaDC 200 mM, the first injection give rise to a high exothermic peak (Figure 5B), followed by small endothermic peaks, related with the demicellization process of the sodium deoxycholate. This behavior indicates a strong specific interaction between pectin and NaC in monomeric form. To better understand the nature of this interaction we have repeated the calorimetric titration by using a NaDC solution 40 mM, as shown in Figure 6. The ITC output suggests a highly exothermic binding between pectin and NaDC at a concentration well below the cmc, where NaDC is in monomeric form. Moreover, also the saturation of the sites present on the fiber is reached at a NaDC concentration well below the cmc, as shown in Figure 6. This behavior is evidence of a specific binding between pectin an NaDC as a monomer. The subtraction of the relatively small endothermic quantity of heat due to the demicellization process and, after the cmc, to the dilution of the micelles, does not change the trend observed. Considering the titration of the pectin with NaDC 200 mM, shown in Figures 5B,B’, where the saturation is reached with the first injection, it is possible to evaluate the value of the cmc (cmc*) in presence of 1 g L^–1^ of pectin by considering the shift of the inflection point in the curve enthalpy vs. bile salt concentration in respect to the titration without pectin (47). The cmc in presence of 1 g l^–1^ of pectin results about 15 mM: this means that 6 mmoles of NaDC per each gram of pectin are subtracted from the solution due to the binding of the monomer to pectin. This quantity is very relevant and indicates a great efficiency of pectin in removing this kind of bile salt. Considering the chemical structure of the NaDC and the behavior of pectin toward the other bile salts considered in this paper, it seems that the lack of one OH group is necessary for the binding but not sufficient. In fact, the conjugation with taurine of the carboxylic group completely hinders the binding (see Figures 5D,D’), so indicating a possible involvement of the carboxylic group in the interaction.

### Bile salts—chitosan interaction

Chitosan is a natural polycationic linear polysaccharide obtained from partial deacetylation of chitin, one of the most widespread naturally occurring polysaccharides (47–51). Chitin, formed by β(1→4)- D- glucosamine units with a variable degree of N-acetylation, is, in fact, the structural element of the exoskeleton of arthropods. Due to its non-toxicity, biodegradability, biocompatibility, low allergenicity and antibacterial properties, chitosan has received considerable attention in biomedical field and in biomedical engineering (48–51). Primary amine groups formed by deacetylation are responsible of the rather special cationic nature of chitosan, being the greatest part of polysaccharides either neutral or negatively charged in acidic environment (48). In stomach, chitosan becomes a soluble salt and reacts with hydrochloric acid, swells and forms a positively charged gel to which negatively charged molecules, i.e., fats, fatty acids, other lipids, and bile acids, strongly bind (52). Chitosan, insoluble at pH 6.3, precipitates in the intestine leading to the inhibition of their absorption and recycling from the intestine to the liver. Also recently, a lot of studies report that chitosan treatment results in significantly lower serum low density lipoprotein (LDL) cholesterol concentrations (52–55).

**FIGURE 7:**
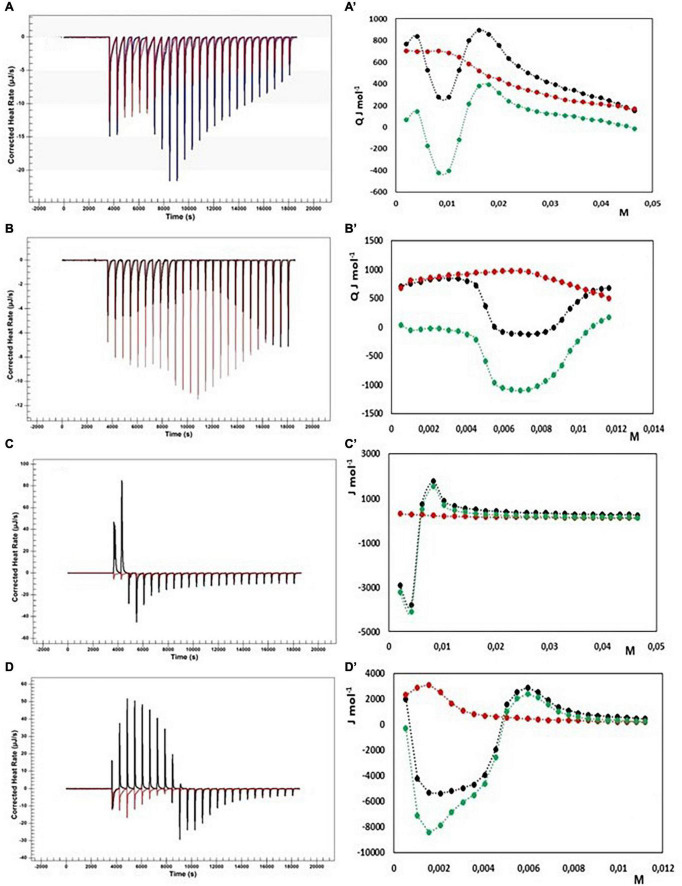
Heat rate (μJ s^–1^) vs. time profiles obtained from 25 injection each of 10 μL into a 960 μL reaction cell filled with a chitosan solution 1 g L^–1^ in 100 mM acetate buffer at pH = 3.0 (black) compared with the dilution of bile salt (red) in the same conditions, and (specified by a single quote mark) the dependence of the enthalpy change vs. bile salt concentration in the reaction cell for: **(A)** NaTC 200 mM; **(B)** NaTC 50 mM; **(C)** NaTDC 200 mM; **(D)** NaTDC 50 μM. Black dots represent the interaction enthalpy between pectin and bile salt, the red dots the dilution enthalpies of bile salt and the green dots their difference.

Due to the polycationic nature of the chitosan we expect a strong electrostatic interaction between bile salts and fiber. Unfortunately, being the fiber soluble only in acidic environment, where NaC and NaDC precipitate, our ITC experiments concern only the interaction with the bile salts conjugated with taurine. We have followed the sample preparation method used by Thongngam and McClements (47) to study the interaction between chitosan and NaTC, so obtaining transparent solutions. Results are shown in Figure 7.

Initially, the cmcs of NaTC and NaTDC in 100 mM acetate buffer at pH = 3 have been evaluated through dilution experiments, resulting lower than in aqueous solution, due to the increasing of the ionic strength associated with the 100 mM acetate buffer (NaTC cmc = 13 mM and NaTDC cmc = 2.4 mM). On the contrary, the changes in enthalpy upon micelle formation remains unchanged, in agreement with literature data (30).

The experiments with NaTC qualitatively agree with the results obtained by Thongngam and McClements (47): after an initial endothermic enthalpy change, the curve in Figure 7A’ shows a deep minimum around 10 mM NaTC, then a maximum around 20 mM is reached with a successive decrease to a constant value. To better examine the behavior at low concentrations of NaTC, we have repeated the measurements by using a more diluted NaTC solution (50 mM in 100 mM acetate buffer at pH = 3, see Figures 7B,B’). If the dilution heat of NaTC is subtracted to the total reaction heat, to have only the heat involved in the interaction between chitosan and NaTC, the heat results very small till a concentration of about 4 mM is reached inside the cell. After that, the reaction becomes exothermic. The exothermicity could be due to the electrostatic interactions between opposite charges, but also to the heat released by micelle formation when the concentration of the monomers bound to the fiber locally reaches the cmc value. The formation of micelles on the fiber give rise, in our opinion to a change of conformation of the fiber itself. Exactly on this matter, our model for the interaction differs a little from that proposed in Munarin et al. (44). NaTC saturates the fiber in his monomeric form and is subtracted from the solution. After saturation a further addition of NaTC absorbs the demicellization heat until its concentration of monomers in solution reaches again the cmc. When chitosan is present, NaTC cmc* results about 19 mM, so that it can be roughly evaluated that 6 mmoles of NaTC are bound per 1 gram of chitosan, not far from the results obtained in Munarin et al. (44) also considering the different batch of chitosan used in the two studies.

ITC titration have been performed in the same conditions also with NaTDC. Substantially the same considerations can be done also for this system, with two relevant differences: NaTDC has a lower cmc than NaTC and a greater Δ*H*_mic_ as absolute value than NaTC. The heat involved in the interaction between chitosan and NaTDC results much greater than that with NaTC. A strong exothermic interaction appears as sun as the NaDC solution is added to the fiber when the concentration of NaDTC is lower than the cmc. A minimum is reached at about 2 mM NaTDC, indicating that NaTDC binds to the fiber as a monomer. After the fiber becomes saturated, a maximum at about 6 mM is observed in the thermogram. Because the monomer formed by the disruption of micelles in the first injection is subtracted to the solution by the binding with the fiber only after saturation we observe the usual trend for the surfactant, i.e., the heat of demicellization till the concentration in solution reaches the cmc, followed by the dilution of micelles. From the shift of curve of NaTDC in presence of fiber and in absence, the quantity of NaTDC bound to the fiber can be approximately evaluated, as for NATC. The cmc* (in presence of chitosan) results 6.5 mM. This means that about 4 millimoles per gram are bound to the chitosan with a strongly exothermic reaction. At least two different contributions must be considered and are evidenced by the shape of the peaks of the first injection and at saturation: the endothermic heat of demicellization and the exothermic electrostatic interaction with the oppositely charged fiber. Moreover, following the model proposed by Thongngam and McClements (47), the chitosan molecules undergo a conformational change allowing the bile salt molecules to stick together, forming micelle like cluster within the chitosan structure. In our opinion, bile salts, once bound to the fiber, feel a local concentration greater than the cmc, notwithstanding the bulk concentration is less than the cmc, and are induced to form micelles. The micelle formation causes a structural change of the linear polymer, as suggested in many studies of polymer surfactant interaction (56–58). Therefore, two more heat effects contribute to the total interaction heats: the heat associated with the change of conformation of the polymer and the heat released in micelle formation. The last one could, at least in part, explain the reason of the much greater exothermic interaction in the case of NaTDC. In any case the addition of both NaTC and NaTDC solutions to the clear solution of chitosan give rise to a turbid solution with the formation of a precipitate.

## Conclusion

Notwithstanding the general acceptance of the healthy role of the fibers in the diet, the mechanism underlying the cholesterol-lowering ability of soluble fibers is still under discussion. Because it is generally accepted that SDF can bind bile salts, preventing their reabsorption, we started a systematic study of the binding ability of some SDF originated from different natural sources with regard to selected bile salts by ITC. This information is also useful to design new functional foods containing a pool of fibers with the best performances in reducing blood cholesterol, fundamental strategy to decrease the chances of a CVD occurrence in the population.

Measurements done on two negatively charged SDF, namely alginate and pectin in water at spontaneous pH, do not show specific binding interaction with BS for alginate. On the contrary, pectin shows a strong exothermic bond with monomer NaDC. Each gram of pectin binds about 6 millimoles of NaDC as it is possible to evaluate from the shift of NaDC cmc in presence of SDF.

Chitosan, positively charged and soluble only at low pH, was studied in 100 mM acetate buffer at pH = 3. It shows, as expected, strong exothermic interactions with the bile salts soluble at this pH (NaTC and NaTDC) with precipitate formation. For NaTC, the exothermic peak starts at about 5 mM. Probably at this concentration the adsorption on the fiber locally reaches the cmc value and micelles start forming on the fiber inducing its conformational change. For NaTDC the same process occurs at much lower concentrations, due to the lower cmc value, and with a greater quantity of heat involved. From the shift of the cmc in presence of chitosan it can be evaluated that about 6 mmoles of NaTC and 4 mmoles of NaTDC can bind to 1 g of chitosan.

This first set of results shows that for some SDF the binding of BS could be an important mechanism in cholesterol lowering but other mechanism, such as the viscosity increase, must be kept into account. The information here presented could be a starting point for the design of functional foods with high cholesterol lowering ability and agreeable taste so that they can be easily consumed both as preventive and therapeutic approach reaching a better compliance within the patients.

## Data availability statement

The original contributions presented in this study are included in the article/supplementary material, further inquiries can be directed to the corresponding author.

## Author contributions

EF and MM: conceptualization and writing. EF and CC: methodology. MM, CC, and EF: review and editing. All authors have read and agreed to the submitted version of the manuscript.
